# The Multiple Sclerosis Health Resource Utilization Survey (MS-HRS): Development and Validation Study

**DOI:** 10.2196/17921

**Published:** 2020-03-17

**Authors:** Nils-Henning Ness, Rocco Haase, Raimar Kern, Dirk Schriefer, Benjamin Ettle, Christian Cornelissen, Katja Akguen, Tjalf Ziemssen

**Affiliations:** 1 University Hospital Carl Gustav Carus Dresden Germany; 2 MedicalSyn GmbH Dresden Germany; 3 Novartis Pharma GmbH Nuremberg Germany; 4 Siemens Healthineers AG Erlangen Germany

**Keywords:** multiple sclerosis, patient-reported outcome measures, resource utilization, validation, questionnaire development

## Abstract

**Background:**

Survey-based studies are frequently used to describe the economic impact of multiple sclerosis (MS). However, there is no validated health resource survey available, preventing comparison of study results and meaningful conclusions regarding the efficiency of long-term treatments.

**Objective:**

The aim of this study was to develop and validate a tablet- and paper-based MS health resource utilization survey.

**Methods:**

We developed and validated the Multiple Sclerosis Health Resource Utilization Survey (MS-HRS), consisting of 24 cost items for paper and tablet users. Data for validation came from two large German observational studies. Survey practicability was assessed according to the response rate. Reliability was described using test-retest reliability as well as Guttman lambda. Construct validity was assessed as convergent and discriminant validity via correlations with associated patient-reported outcomes and known-group analyses.

**Results:**

In total, 2207 out of 2388 (response rate: 92.4%) patients completed the survey and were included to determine psychometric properties. The test-retest reliability had an intraclass correlation coefficient of 0.828 over a course of 3 months. Convergent validity analyses showed that total costs correlated positively with increased disability (*r*=0.411, *P*<.001). For discriminant validity, correlations of total costs with the Treatment Satisfaction Questionnaire for Medication ranged from −0.006 (convenience) to −0.216 (effectiveness). The mean annual cost was €28,203 (SD €14,808) (US $39,203; SD US $20,583) with disease-modifying therapies.

**Conclusions:**

The MS-HRS is a multilingual, reliable, valid, electronically available, and easy-to-administer questionnaire providing a holistic cross-sectional and longitudinal assessment of resource utilization in patients with MS.

## Introduction

Multiple sclerosis (MS) is a potentially severe cause of neurological disability throughout adult life, leading to many years with high economic burden of the disease [1]. Studies on resource utilization in patients with MS have analyzed secondary data, such as administrative data of health insurance or health care providers, which have several strong limitations [2]. First, data accuracy may not be sufficient as several health care services can, but should not, be combined under one capitation. Second, the societal perspective cannot be considered, as only a share of all costs is refunded. In complex diseases like MS, patients’ needs exceed the scope of primary and secondary health care providers, making a societal perspective even more important [3]. Third, billing data do not include important clinical data, making it impossible to determine the reasons for cost increases in the investigated population.

As another approach, diaries are commonly used to prospectively gather information on patient-level data [3]. However, the challenges in using diaries are thoroughness in reporting and high drop-out rates in time periods longer than 1 year [4].

Questionnaire-based cross-sectional studies represent a third well-recognized way of cost assessment in MS research, and they are well suited to analyze the occurrences of certain utilization behaviors [4-11]. Such surveys have the potential to include all relevant cost dimensions and may be applied to several stakeholders of the health care process. For instance, disease-modifying therapies (DMTs) are the main cost drivers for patients in earlier disease stages, and indirect costs are mainly responsible for the economic burden in later disease stages. However, cross-sectional investigations cannot assess the temporal associations between an intervention and an outcome. Such objectives require longitudinal data, which are fundamental for health economic evaluations, such as cost-effectiveness studies. Despite the long tradition of health economic evaluations in MS, there is no validated questionnaire with sufficient psychometric properties available, preventing comparison of study results between populations and meaningful conclusions [12,13].

The aim of our work was to develop and validate an easy to administer questionnaire that provides a holistic longitudinal assessment of resource utilization for clinical practice and elaborate research approaches.

## Methods

### Questionnaire Development

**Figure 1:**
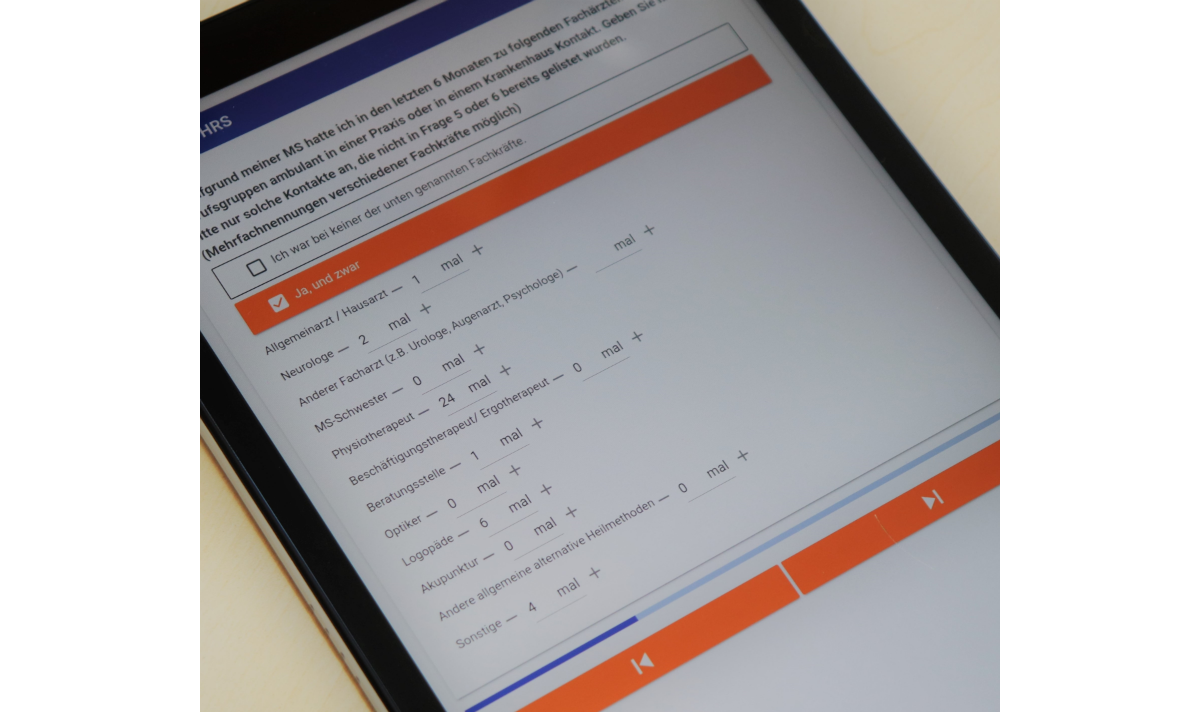
Image of the tablet app of the German version of the Multiple Sclerosis Health-Resource Utilization Survey.

A German expert group consisting of neurologists, health care administrators, psychologists, and MS nurses developed the first version of the Multiple Sclerosis Health Resource Utilization Survey (MS-HRS) at the University Hospital Dresden in 2009. Following intensive feedback from physicians, nurses, and patients, a second more time-efficient version was created in 2016. The requirements from clinical practice, such as time-saving use in everyday clinical practice, updatable pricing, and avoidance of double documentation, were decisive for the development of the digital version, which is browser-based and can be used across most devices (Figure 1). The time-saving use was realized through an adaptive questionnaire structure by presenting only the necessary items electronically.

The current digital German and translated English versions of the MS-HRS are in line with the recommendations on the core elements of a standardized resource questionnaire and can be completed in 10 to 15 minutes [14].

Health economic analysis can be performed from different perspectives with the societal perspective being the broadest [15]. For the practical evaluation of costs, three steps are considered, namely identification of resource consumption, quantification of resource use, and valuation of resources [15].

### Identification of Resource Consumption

Information on resource consumption was obtained from studies, guidelines, textbook knowledge, administrative and accounting data, and expert opinions [4,8,16,17].

Costs were classified into direct medical, direct nonmedical, and indirect costs, according to German health technology assessment guidelines. Keeping multiple use cases in mind, we divided the tool into a core and an additional set of items (Table 1). The core elements are constitutive for the health resource utilization model and sufficient to capture the scope of resource utilization, whereas the additional questions foster the understanding of resource utilization.

**Table 1:** Core items of the Multiple Sclerosis Health Resource Utilization Survey: content and response option.

Domain (number of questions) and question label		Item	Response option	
**Direct medical costs (5)**				
	Inpatient stays	Stays in a hospital, especially in a neurological ward, rehabilitation clinic, and nursing home	Number of stays	
	Outpatient stays	Stays in a hospital, especially in a neurological ward, rehabilitation clinic, and nursing home	Number of stays	
	Professional consultations	Contact with neurologists, other specialists, multiple sclerosis nurses, physiotherapists, occupational therapists, counselling centers, opticians, speech therapists, acupuncturists, other alternative health care professionals, and others	Number of contacts	
	Examinations	Examinations undergone: magnetic resonance imaging, computed tomography, lumbar puncture, and blood examinations	Number of examinations	
	Over-the-counter medication		Expenditure in Euro	
	Medical consumables		Expenditure in Euro	
	Professional care	Assistance from professional caregiver and home help	Number of hours per week	
**Direct nonmedical costs (2)**				
	Informal care	Assistance from friends and family members	Number of hours per week	
	Investments and purchases	Investments and purchases: housing, car modifications, walking aids, manual wheelchair, automatic wheelchair, scooter, bed, and others	Expenditure in Euro	
**Indirect costs (3)**				
	Employment	Total hours of working time per week	Number of hours	
	Employment: Sick leave	Days of sick leave	Number of days	
	Employment: Presenteeism	Extent of reduced productivity at work	Likert scale (0-10)	
	Employment: Disability pension	Extent of disability pension	Percentage	

### Quantification of Resource Use

We used the most accurate method of microcosting defined as “direct enumeration and costing out of every input consumed in the treatment of a particular patient” to quantify resources (Table 2) [18]. Recall periods of resource utilization have been determined to avoid over- and underreporting. Longer recall periods lead to telescoping effects, in which events occurring outside the time frame are mistakenly included in the intended period [19]. Furthermore, we took into account that more frequent events and those that are less salient are less likely to be recalled accurately over a long period. In conclusion, the recall period may differ with respect to the nature of cost items but should never exceed 12 months. We recommend intervals for the assessment between 3 and 6 months. It is noticeable that unified recall periods across all items of the survey made the questionnaire more intelligible for patients. In our results, we report all calculated costs as per quarter.

**Table 2:** Health resources and quantification.

Domain		Item	Valuation
**Direct medical costs**			
	Inpatient care/day admission	Days in hospital, rehabilitation, and nursing wards	Standardized cost units
	Ambulant consultations	Visits to general practitioners, neurologists, others specialists (urologists, ophthalmologists, and psychiatrists), multiple sclerosis nurses, physical therapists, psychologists, occupational therapists, opticians, speech therapists, acupuncturists, and other alternative healing professionals	Standardized cost units
	Investigations/diagnostics	Magnetic resonance imaging, computed tomography, spinal tap, blood tests, and others	Physicians’ fee schedule
	Over-the-counter medication	Medication and recommending doctor	Patient reported
	Disease-modifying therapies		Annual therapy costs as calculated from medication reports
	Home help and support of professionals	Professional help, household care, and personal assistance	Human capital approach
	Medical consumables	Medical consumables	Patient reported
**Direct nonmedical costs**			
	Investments and equipment	House and car modifications, walking aids, wheelchair (manual and electric), scooter, sickbed, and others; payer and grants	Patient reported
	Informal care	Time taken for preparation of meals, climbing stairs, personal care, drug administration, transport, and others; reduction of working hours of relatives	Opportunity costs method
**Indirect costs**			
	Employment and labor productivity	Full or part time work, sick leaves, reduced working time, change of work, and loss of earning	Human capital approach
	Employment and labor productivity	Absence hours (multiple sclerosis and others), total working hours, and productivity scale	Human capital approach

### Valuation of Resources

Evaluations conducted from societal perspectives are intended to reflect societal opportunity costs, which are equal to market prices in perfectly competitive markets. Nevertheless, markets in the health care sector are imperfect owing to statutory regulations. Hence, societal opportunity costs have to be approximated in most cases. Therefore, data from Bock et al were used wherever available (Table 3) [20].

Owing to a lack of data, few valuations were calculated from existing values. Visiting a nurse was rated with the lowest monetary value of €16.42. Furthermore, psychologist contact was monetarily valued as visiting a psychotherapist. The valuation for other specialists (eg, psychiatrist, urologist, and optician) was calculated as the mean of the given examples. Societal opportunity costs for investigations were approximated from the physicians’ fee [21].

To calculate annual costs for DMTs, we used defined daily dose net costs multiplied by 365 days [22]. Net costs account for statutory manufacturer discounts as well as pharmacy discounts (Table 4). The work productivity loss (absenteeism, early retirement, and presenteeism) was calculated using the human capital approach [23]. Absenteeism was defined as not showing up for work, whereas presenteeism was defined as reduced work productivity due to health problems. The loss resulted from the total number of lost hours multiplied by the average salary per hour. Any hour not worked was considered as lost. Data for this calculation were obtained from official statistics [24].

In 2011, 233 working days were used for the calculation, and the average number of working hours was 1406.2 hours [25]. Additionally, the average hourly labor cost was €29.90 [20]. The monetary work productivity loss due to sick leave was calculated as follows:

Productivity loss = (1406.2 working hours/233 working days) × €29.90 hourly salary

Occupational disability was calculated as the product of daily wage, average number of working days, and percentage of disability, with a maximum of €42,045.38 per year. The maximum hours dedicated to informal care was set to 60 hours per week according to German policies.

To ensure comparability of cash flows, prices from different periods were adjusted to the 2011 price level using the general price index for the national economy. This applies to patients’ self-reported medications, investments, medical consumables, and DMTs. Consumption of the remaining resources was valued with prices from the year 2011 to generate comparable costs within our validation population. For other purposes, more recent values may be derived by applying a conventional cost inflation of 2%.

All costs are reported in Euro. In 2011, the Euro to US dollar annual average exchange rate was equal to 1.392.

**Table 3:** Resource valuation per unit.

Cost category		Monetary valuation
**Direct medical costs (inpatient)**		
	Hospital	€593.04
	Rehabilitation	€121.85
	Nursing	€69.80
**Direct medical costs (outpatient)**		
	Hospital	€385.48
	Rehabilitation	€46.68
	Nursing	€46.15
**Direct medical costs (ambulant consultations)**		
	General practitioner	€20.06
	Neurologist	€44.72
	Other specialists	€34.73
	Nurse	€16.42
	Physiotherapist	€16.42
	Psychologist	€78.08
	Occupational therapist	€37.51
	Optician	€34.78
	Speech therapist	€38.59
	Acupuncturist	€18.24
	Other alternative healing professionals	€27.40
**Direct medical costs (investigations/diagnostics)**		
	Magnetic resonance imaging	€120.21
	Computed tomography	€73.78
	Lumbar puncture	€38.90
	Blood tests	€1.10
	Others	€60.66
Direct medical costs (over-the-counter medication)		Patient reported
Direct medical costs (disease-modifying therapy)		See Table 4
Direct medical costs (medical consumables)		Patient reported
Direct medical costs (home help/professional care)		€27.57
**Direct nonmedical costs**		
	Equipment, aids, and modifications	Patient reported
	Informal care	€21.09
**Indirect costs**		
	Sick leave	€180.45
	Disability pension (full year)	€42,045.38

**Table 4:** Costs of disease-modifying therapies per year (in €).

Year	AVO^a^	REB^b^	BET^c^	EXT^d^	COP^e^	GIL^f^	TYS^g^
2010	18069.27	22626.46	17977.14	15528.45	16623.45	N/A^h^	24625.10
2011	18611.35	23305.25	18516.45	15994.30	17122.15	26698.58^i^	25363.85
2012	18182.19	22900.46	18132.07	15604.68	16377.93	25907.56	24586.58
2013	17981.00	22765.35	17815.29	15431.93	16197.00	22571.44	24214.41
2014	19437.31	24432.98	17601.95	15441.48	17035.61	18965.36	26194.92
2015	19333.96	24155.25	17517.70	15370.25	16733.32	20393.73	24280.75
2016	19247.42	23987.27	17384.09	14680.25	16648.48	21516.72	23321.06
2017	18865.10	23529.38	17047.81	15715.49	16352.26	20784.68	22902.02

^a^AVO: Avonex.

^b^REB: Rebif.

^c^BET: Betaferon.

^d^EXT: Extavia.

^e^COP: Copaxone.

^f^GIL: Gilenya.

^g^TYS: Tysabri.

^h^N/A: not applicable.

^i^As listed in the technology assessment report.

### Study Population

Patients with relapsing-remitting MS (RRMS) were recruited in two prospective noninterventional multicenter studies conducted in Germany [26,27]. In that context, assessments of medical history and other general data, such as the Expanded Disability Status Score (EDSS), were performed by the treating neurologist. The current analyses are limited to patients with an EDSS of 0-6.0 to ensure sufficiently large subpopulations. Approval for both studies was obtained from independent local ethics committees, and all patients provided written informed consent for the collection of data [26,27].

### Practicability and Reliability

Practicability was determined by the response rate of patients completing the MS-HRS. In addition, we compared the characteristics of completers and noncompleters to avoid selection bias. For reliability and validity analyses, we focused on the core elements of the survey. As we did not intend to evaluate DMTs at this step, costs for DMTs were not part of our methodological evaluation, which focused on psychometric properties.

For reliability analysis, test-retest reliability of the total costs in a group of stable patients over 3 months was estimated. Stable patients were defined as those not having relapse or increase in the EDSS during the 3 months of the retest period and another 3 months prior to the assessment. Thresholds for intraclass correlation coefficient (ICC) were applied as recommended (ICC<0.5: poor reliability; 0.5≤ICC<0.75: moderate reliability; 0.75≤ICC<0.9: good reliability; and ICC>0.90: excellent reliability) [28]. Guttman lambda 2 and lambda 6 are reported for the monetarized standardized items of the health resource utilization model with respect to the heterogeneous structure, providing a lower bound estimate of the consistency of the pricing approach. In an ordinary setting for test construction, desired levels would lay above 0.7. As our survey is not based on latent constructs but instead on real costs, consistency analysis is not part of the primary evaluation of reliability, but it provides additional insights for the model and the cost components.

### Validity

Repeated expert consensus meetings of health economists, psychologists, and neurologists were conducted to secure face validity in terms of consistency and completeness.

Construct validity was assessed as convergent and discriminant validity via correlations with associated patient-reported outcomes (PROs) and known-group analyses by the EDSS (ranges: 0-1.0, 1.5-2.5, 3.0-4.0, and 4.5-5.5). The selected PRO measures were the EuroQol-5 Dimensions (EQ-5D), UK Neurological Disability Scale (UKNDS), and Patient Reported Outcome Indices for MS (PRIMUS) [29-32]. We expected to find significantly higher costs in groups with higher EDSS scores and correlations above 0.40 for convergent validity. As we did not intend to evaluate DMTs, the Treatment Satisfaction Questionnaire for Medication (TSQM) should present correlations clearly below 0.3 for discriminant validity [33].

### Statistical Analysis

Continuous values are reported as arithmetic mean and SD. Ordinal values are reported as median and IQR. One-way random ICCs were used to calculate estimates for the test-retest reliability. All other correlations were calculated with Spearman rank correlation coefficients. Kruskal-Wallis H tests were conducted for known group analyses. Mann-Whitney U tests with adjustments by Bonferroni correction for multiple tests were applied for pairwise comparisons. All reported *P* values were compared to an alpha error level of 5%. No imputations were made for missing values.

## Results

### Patient Characteristics

In total, 2207 of 2388 patients completed the questionnaire at baseline and were therefore included in the validation process. The study population had a mean age of 41.73 (SD 10.19) years and was mostly female (1609/2207, 72.90%) (Table 5). Employed patients (1347/2207, 61.03%) were working predominantly full time (794/1347, 58.95%).

In terms of relapses within the previous year, active (1015/2207, 45.99%) and nonactive (1192/2207, 54.01%) patients were balanced in the population. Participants reported a mean disease duration since diagnosis of 7.54 (SD 6.11) years and a mean EDSS of 2.43 (SD 1.57).

**Table 5:** Characteristics of the study population (N=2207).

Parameter			Value
**Age, years**			
	Mean (SD)	41.73 (10.19)	
	Median (IQR)	42.00 (34.00-49.00)	
**Gender**			
	Female, n (%)	1609 (72.90%)	
**Employment**			
	Employed, n (%)	1347 (61.03%)	
	Full-time employed, n (%)	794 (58.95%)	
**Number of relapses in the previous year, n (%)**			
	0	1192 (54.01%)	
	1	612 (27.73%)	
	2	267 (12.10%)	
	≥3	98 (4.44%)	
	Unknown	38 (1.72%)	
**Duration of disease since diagnosis, years**			
	Mean (SD)	7.54 (6.11)	
	Median (IQR)	6.00 (3.00-11.00)	
**EDSS^a^**			
	Mean (SD)	2.43 (1.57)	
	Median (IQR)	2.00 (1.00-3.50)	

^a^EDSS: Expanded Disability Status Score.

### Validation of the Questionnaire

#### Practicability

Looking at the number of patients completing the questionnaire, a good response rate of 92.4% (2207/2388) was achieved. Therefore, the responses of 2207 patients could be used to calculate total costs and all other parts of the health resource model. Noncompleting patients (n=181) did not differ in their gender distribution, age, or EDSS, but presented a slightly longer disease duration (mean 9.67 [SD 7.21] years, *P*<.001).

#### Reliability

Reliability was mainly assessed as test-retest reliability in a stable subgroup of patients. Overall, 1192 of 2207 (54.01%) patients fulfilled the criterion of presenting stable MS within that period. The ICC for this group over a course of 3 months was 0.828. In addition, Guttman lambda 2 (λ2=0.679) and lambda 6 (λ6=0.694) indicated an acceptable consistency between the standardized monetarized items of the health resource model (excluding DMT costs).

#### Validity

We analyzed the construct validity for total costs of the MS-HRS via known groups (excluding DMT costs). In all four EDSS groups, we found significantly different MS-related total costs and subcosts (all *P*<.001; Table 6). Further, all pairwise comparisons indicated significant differences in the direction as expected before (all *P*<.001 for total costs, all *P*<.05 for subcosts; Table 6).

**Table 6:** Known-group analysis: health resource utilization costs of patients with multiple sclerosis per quarter by disability (N=2059).

Parameter	EDSS^a^ 0-1.0 (n=562)			EDSS 1.5-2.5 (n=756)			EDSS 3.0-4.0 (n=589)			EDSS 4.5-5.5 (n=152)		
	Mean	SD	Median	Mean	SD	Median	Mean	SD	Median	Mean	SD	Median
Total cost (€)	1099	2211	150	2295	3647	585	3112	3979	1334	4733	4639	4158
Direct medical cost (€)^b^	309	910	99	541	1724	165	789	1998	249	1003	2295	411
Direct nonmedical cost (€)	12	108	0	20	154	0	45	152	0	158	428	0
Indirect cost (€)	777	1822	0	1734	2820	0	2280	3178	127	3572	3572	3637

^a^EDSS: Expanded Disability Status Score.

^b^Disease-modifying therapies are not included in direct medical cost.

Convergent validity analyses showed that total costs correlated positively with increased (patient-reported) disability (UKNDS sum score: *r*=0.411) and lost ability to participate in daily routines and activities (PRIMUS Activities: *r*=0.423) and negatively with health-related quality of life (PRIMUS QoL: *r*=0.350; EQ-5D: *r*=−0.342) (all *P*<.001).

For discriminant validity, correlations of total cost with TSQM scores ranged from −0.006 (convenience) to −0.216 (effectiveness). As expected, the inclusion of DMT costs lowered the correlations with all PROs (by 0.07 on average), but the relations between correlations were maintained.

### Resource Utilization

Majority of patients stated that they used direct medical services in the past 3 months (Table 7). In contrast, less than every second patient (44%) had indirect medical costs and 16% had direct nonmedical costs. Besides DMTs, indirect costs were the main cost drivers ahead of direct medical and direct nonmedical costs.

Patients were mainly treated in private practices (2068/2207, 85%) and less often during inpatient hospital stays (138/2207, 6%) and day care admissions (65/2207, 3%). However, the highest cost was for inpatient treatments (€315.06 [SD €1587.09]), followed by consultations in the primary sector (€209.87 [SD €292.73]) and day admissions in hospitals (€32.22 [SD €311.68]). Total costs per quarter averaged €2462 (SD €3650; median: €631) without DMTs and €7126 (SD €3697; median: €5871) with DMTs. Therefore, annual costs for mild-to-moderate RRMS ranged between €9528 (SD €14,603) without DMTs and €28,203 (SD €14,808) with DMTs.

**Table 7:** Health resource utilization costs in patients with multiple sclerosis per quarter (N=2207).

Variable		Value^a^
**Direct medical costs (without disease-modifying therapies)**		
	Users, n (%)	2068 (93.70)
	Mean (SD)	601.30 (1708.55)
	Median (IQR)	174.00 (75.00-417.50)
**Inpatient care**		
	Users, n (%)	138 (6.25)
	Mean (SD)	315.06 (1587.09)
	Median (IQR)	0.00 (0.00-0.00)
**Day admission**		
	Users, n (%)	65 (2.95)
	Mean (SD)	32.22 (311.68)
	Median (IQR)	0.00 (0.00-0.00)
**Consultations**		
	Users, n (%)	1817 (82.33)
	Mean (SD)	209.87 (292.73)
	Median (IQR)	109.50 (44.72-268.32)
**Examinations**		
	Users, n (%)	1429 (64.75)
	Mean (SD)	32.34 (35.63)
	Median (IQR)	30.05 (0.28-45.77)
**Over-the-counter medication**		
	Users, n (%)	693 (31.40)
	Mean (SD)	15.02 (46.43)
	Median (IQR)	0.00 (0.00-10.00)
**Professional care**		
	Users, n (%)	115 (5.21)
	Mean (SD)	7.02 (44.79)
	Median (IQR)	(0.00-0.00)
**Disease-modifying therapies**		
	Users, n (%)	2185 (99.00)
	Mean (SD)	4733.24 (820.30)
	Median (IQR)	4629.11 (4280.54-5656.61)
**Direct nonmedical costs**		
	Users, n (%)	352 (15.95)
	Mean (SD)	44.65 (229.51)
	Median (IQR)	0.00 (0.00-0.00)
**Investments**		
	Users, n (%)	38 (1.72)
	Mean (SD)	9.14 (165.80)
	Median (IQR)	0.00 (0.00-0.00)
**Informal care and community service**		
	Users, n (%)	337 (15.27)
	Mean (SD)	37.25 (158.57)
	Median (IQR)	0.00 (0.00-0.00)
**Indirect costs**		
	Users, n (%)	977 (44.27)
	Mean (SD)	1816.50 (2880.74)
	Median (IQR)	0.00 (0.00-3037.00)
**Short-term absence**		
	Users, n (%)	320 (14.50)
	Mean (SD)	441.53 (1782.18)
	Median (IQR)	0.00 (0.00-0.00)
**Disability pension**		
	Users, n (%)	352 (15.95)
	Mean (SD)	852.98 (2062.98)
	Median (IQR)	0.00 (0.00-0.00)
**Presenteeism**		
	Users, n (%)	475 (21.52)
	Mean (SD)	522.00 (1333.18)
	Median (IQR)	0.00 (0.00-0.00)

^a^All costs are in Euro.

## Discussion

### Principal Findings

The MS-HRS represents a reliable, valid, and easy-to-administer questionnaire providing a holistic assessment of resource utilization for patients with MS. Health resources were derived from all pathways of patients with MS in an adapted health resource model for MS.

Some very respectable efforts have already been taken to research the health economic footprint of MS [2,5-7]. In Europe, Kobelt et al repeatedly assessed the costs and burden of MS in a cross-sectional survey approach, including direct, indirect, and intangible costs [6,7]. Concepts and definitions of subcosts may differ over time with respect to scope and style of reporting (eg, intangible costs where a clear line is recommended) [34]. For our model approach, we gave strong emphasis to direct and indirect costs, as done by Karampampa et al and Reese et al in their models [5,11]. We increased the depth of the assessment of indirect costs by adding a quantification of costs for presenteeism in addition to costs for absenteeism (sick leave and disability pension).

**Figure 2:**
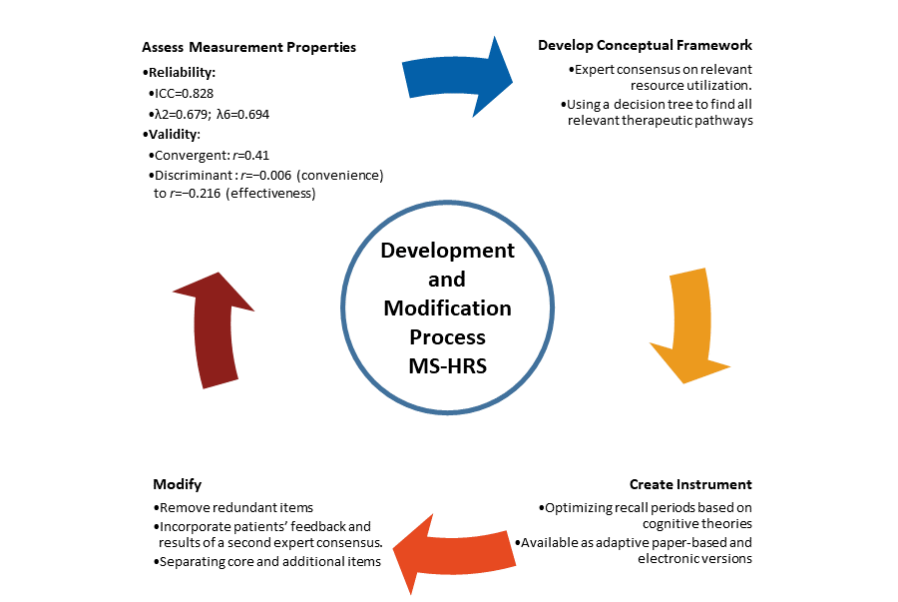
Development and modification process of the Multiple Sclerosis Health-Resource Utilization Survey. ICC: intraclass correlation coefficient; λ2: Guttman lambda 2; λ6: Guttman lambda 6.

Part of our questionnaire development was a validation process that confirmed reliability and validity in a way that no previous approach did within the MS domain (Figure 2). Kobelt et al also recognized the need for validation, but a systematic approach beyond the aspects of practicability and face validity remained a task to fulfil [35]. Even though we did not develop a one-dimensional questionnaire for a latent construct, we were able to demonstrate essential psychometric qualities in the health economic assessment of MS. As of today, a multidomain open-access database of resource-use questionnaires does not contain a published instrument for MS [12]. Most of the cost assessments for MS were not developed to be published for general use but developed for application in certain cross-sectional frameworks. A very recent analysis of real-life cost outcomes underlined the rising interest in longitudinal assessment of health resource utilization [10].

The proportion of patients claiming certain health services was slightly lower in the study by Kobelt et al than in this study [6]. The lower proportion of patients taking over-the-counter medications and investments was particularly noticeable. This may be explained by the less severe disease progression beyond a RRMS profile. Taking this into account, the average annualized disease burden was within the range expected from previous publications [5,7,11,36].

Kobelt et al have recently reported precise price tags for most unit costs, and other authors have at least provided an indirect description of the valuation process and its exact results [5,9]. Differences were found for the valuation of subcosts, such as inpatient hospitalization, owing to different sources of valuation or different definitions for health resource units. In any case, precise and fully transparent reporting of per unit costs is highly recommended, especially for main cost drivers.

Event-related costing can also be considered to be of interest, but a clear separation from costs being assessed via routine data collection is necessary [5]. Otherwise, costs may double due to double assessment. We discussed whether to collect data for both sick days and missed working hours, as described by Reilly et al [16]. Furthermore, asking about sick hours would have meant switching to a recall period of 7 days, whereas the remaining questionnaire covers the last 6 months. Owing to higher usability, we decided to assess sick days only.

Claims data were considered for the assessment of criterion validity. However, there is a disadvantage that only billing-relevant data are available, preventing consideration of a societal perspective. Information on informal care, over-the-counter medication, and presenteeism and partial information on investments and medical consumables is not recorded. In addition, claims data do not provide the number of physician visits by a patient in Germany. In consequence, claims data were not suitable for the validation process.

Health economic studies often require both clinical and economic data. The MS-HRS can easily be used as part of clinical interventional and noninterventional studies to collect economic data. In a large population, we demonstrated that the questionnaire is easy to administer and has good psychometric properties. These characteristics provide the necessary prerequisites for high-quality health economic studies (eg, cost-effectiveness analyses).

### Limitations

A patient-centered questionnaire is subject to notable limitations. Reliable recall periods of health resource use are time-limited, especially in patients who are cognitively more affected by the disease. Furthermore, patients may not want to disclose socioeconomic and health economic information because it is considered too confidential. We did not include information about more recent therapies as we did not gather related data for validation, which will be done in follow-up studies. Beyond this, further costs may be thinkable but less likely to have an impact on a societal level (eg, crowd-funded therapies at the current level and cost-related voluntary work loss). In addition, price tags for cost components have to be updated and adapted to local levels. For patients with progressive MS and patients with severe disability, further studies have to confirm the given psychometric properties.

### Conclusions

The MS-HRS is a promising option to measure costs precisely in cross-sectional and longitudinal settings instead of estimating them or using surrogates. Further country-wise cost weights will facilitate the transparent estimation of MS-related costs across multiple regions. The MS-HRS is available online [37].

## Abbreviations

DMT: disease-modifying therapy
EDSS: Expanded Disability Status Score
EQ-5D: EuroQol-5 Dimensions
ICC: intraclass correlation coefficient
MS: multiple sclerosis
MS-HRS: Multiple Sclerosis Health Resource Utilization Survey
PRIMUS: Patient Reported Outcome Indices for Multiple Sclerosis
PRO: patient-reported outcome
RRMS: relapsing-remitting multiple sclerosis
TSQM: Treatment Satisfaction Questionnaire for Medication
UKNDS: UK Neurological Disability Scale
